# Use of Preventable Risk Integrated Model on behavioral risk factors: a scoping review and bibliometric analysis

**DOI:** 10.3389/fnut.2025.1572234

**Published:** 2025-05-09

**Authors:** Antonio Castillo-Paredes, Gerson Ferrari

**Affiliations:** Escuela de Ciencias de la Actividad Física, el Deporte y la Salud, Universidad de Santiago de Chile (USACH), Santiago, Chile

**Keywords:** behavioral risk factor surveillance system, non-communicable diseases, Preventable Risk Integrated Model, health policy, planning and management, socioeconomic factors, disease prevention

## Abstract

**Introduction:**

Behavioral or habit-based risk factors lead to the development of non-communicable diseases, causing early deaths, disability due to these diseases, and high economic burdens on the public or private health system. The use of the Preventable Risk Integrated Model through the creation of counterfactual scenarios could simulate in the future the number of deaths that can be delayed or prevented if one or more risk factors associated with diet, tobacco, alcohol, or physical activity are modified.

**Objective:**

The objective of this research was to explore the scientific evidence available on the use of the Preventable Risk Integrated Model, identifying the main findings, the most productive authors on the subject, the most used keywords, the countries of origin of the research, and the scientific journals where these studies are published.

**Methods:**

Through the development of four research questions, a search strategy was established for the development of the scoping review and bibliometric analysis of the information obtained.

**Results:**

A total of 24 articles were identified that used the Preventable Risk Integrated Model, which were available in their titles and research abstracts. Regarding the results obtained, this model was used on topics related to food consumption, food composition, nutrient intake, and prevention of non-communicable diseases by reducing some nutrients related to sodium or saturated fats, in addition to the creation of new models supported by the Preventable Risk Integrated Model. In addition, the authors who research the topic, the use of keywords, the countries that have done the most research on the subject, and indexed journals were identified.

**Discussion:**

The studies analyzed focus on recommendations proposed by non-governmental organizations and national policies of each country, while other studies focus on the areas of reduction of death, reduction of costs associated with the prevention of non-communicable diseases and other studies provide information on the creation of the model and another related to the use in other behavioral habits. In addition, networks are visualized for the follow-up of authors doing research on the use of this model.

**Conclusion:**

Finally, the use of the model allowed the projection of preventable deaths or those that could be delayed when modifying the risk factors that cause non-communicable diseases; however, caution must be taken in its proper use due to the consultation of various sources such as databases, use of national surveys, or international information repositories.

**Systematic Review Registration:**

Identifier: DOI 10.17605/OSF.IO/WYNHJ.

## 1 Introduction

Non-communicable diseases (NCDs) are mainly caused by behavioral risk factors that people develop throughout their life cycles (1). These behavioral risk factors are defined as behavioral aspects that generate a risk for the person because they are related to their daily life habits, mainly in diet, planned physical activity (exercise), tobacco consumption, and alcohol consumption, among other habits that, due to their constant presence in their activities, could generate effects for the development of a disease, being able to be innate or inherited (2–10).

These behavioral risk factors related to the intake of ultra-processed foods have an impact on the development of obesity and cardiometabolic risk (11). In addition, the acquisition of an unhealthy diet is a causal factor in the development of NCDs, including obesity, cardiovascular diseases, and type 2 diabetes (12). On the other hand, lack of physical activity or planned physical activity and also sedentary behavior are positively associated with the development of obesity in individuals (13). Indeed, the combination of these two behavioral risk factors raises the probability of becoming cardiovascular risk factors (14). On the other hand, the combination of tobacco use and alcohol consumption is a risk factor for the development of metabolic diseases and consequently, the development of NCDs associated with them, such as the development of cancers, cardiovascular diseases, hypertension, and respiratory diseases (7, 15, 16).

Consequently, the combination of these behavioral risk factors develops specific diseases (17, 18), such as cardiovascular diseases (19, 20), diabetes (21, 22), different types of cancer (22–24), lung diseases (25, 26), liver diseases (27–29), and pancreatic diseases (30, 31). All these diseases, caused by these behavioral risk factors and combined, generate a large number of deaths at an early age (32, 33). However, the economic costs of the prevention and treatment of these diseases are highly expensive for public health (34–38) and can be prevented through models that estimate changes in behavioral risk factors such as dietary habits, physical activity, tobacco, and alcohol in a specific population (39, 40).

The Preventable Integrated Risk Model (PRIME) is a tool that allows modeling scenarios to estimate the number of lives that can be saved through policies that drive countries to change behaviors related to diet, physical activity, and tobacco and alcohol consumption in a particular population. The PRIME model relates behavioral risk factors to NCD mortality, measured directly or indirectly by body mass index, blood cholesterol, or blood pressure (41). This model was created by researchers at the WHO Collaborating Centre on Approaches to NCD Prevention at the University of Oxford to model the impact of national policies on NCD mortality (42). However, it is necessary to explore and check the possible uses in research using the PRIME model; thus, the development of a scoping review (43), which allows us to explore and synthesize the scientific evidence and the use given to it. In addition, in combination with the use of VOS viewer in the development of bibliometric reviews (44), it will make it possible to visualize the most cited authors, use of keywords, countries, and scientific journals where PRIME research is published. To date, no such reviews on the subject have been identified. For this reason, our research objective is to explore the scientific evidence available on the use of the PRIME, identifying the main findings, the most productive authors on the subject, the most used keywords, the countries of origin of the research, and the scientific journals where these studies are published.

## 2 Materials and methods

For the development of this research, two methodologies were used for its development and better understanding (45, 46), which are described below.

### 2.1 Review methods

Scoping review: This methodology was used because it allows researchers to broadly explore the development of a particular area of study and the scientific evidence on, in this case, the use of the PRIME model. For the development of this section, we used the Preferred Reporting Items for Systematic Reviews and Meta-Analyses (PRISMA) Extension for Scoping Reviews (PRISMA-ScR) (43). In addition, it was registered in the Open Science Framework (OSF) https://osf.io/wynhj/ (Supplementary Material 1).

Bibliometric review: For the development of this methodology, the authors who published the most on the subject, keywords used, countries of origin, and scientific journals where the research was published were considered to carry out the analysis. In addition, to carry out the analysis of the articles, Microsoft Excel® and VOSviewer version 1.6.20, (Centre for Science and Technology Studies, Leiden University, Leiden, the Netherlands) were used (44).

#### 2.1.1 Data analysis

The identified data were extracted in two different formats: plain text and Excel. The Excel document was used to perform a descriptive analysis of the results using a Microsoft Excel spreadsheet (v. 2006, Microsoft Corporation, Redmond, WA, USA). The plain text archive allows analysis by co-authorship by authors, co-authorship by organizations, occurrence by keywords, citation by documents, citation by journals, citation by countries, and citation by authors with the VOS viewer program (v.6.19., Center for Science and Technology Studies, the Netherlands).

### 2.2 Research questions

For the development of the research question, the proposal developed by Kart and Kart (47) was used, which is supported by previously carried out study (48, 49), which consists of the statement of the research question to obtain results and subsequent analysis. The combination of both analytical methodologies allowed us to establish the following research questions: What is the scientific evidence on the use of the Preventable Risk Integrated Model? Is there extensive research on the subject? What are the keywords used? Which participating countries publish the most on the subject? Are there specialized journals in the area where this topic is published?

### 2.3 Information search strategies

**Figure 1:**
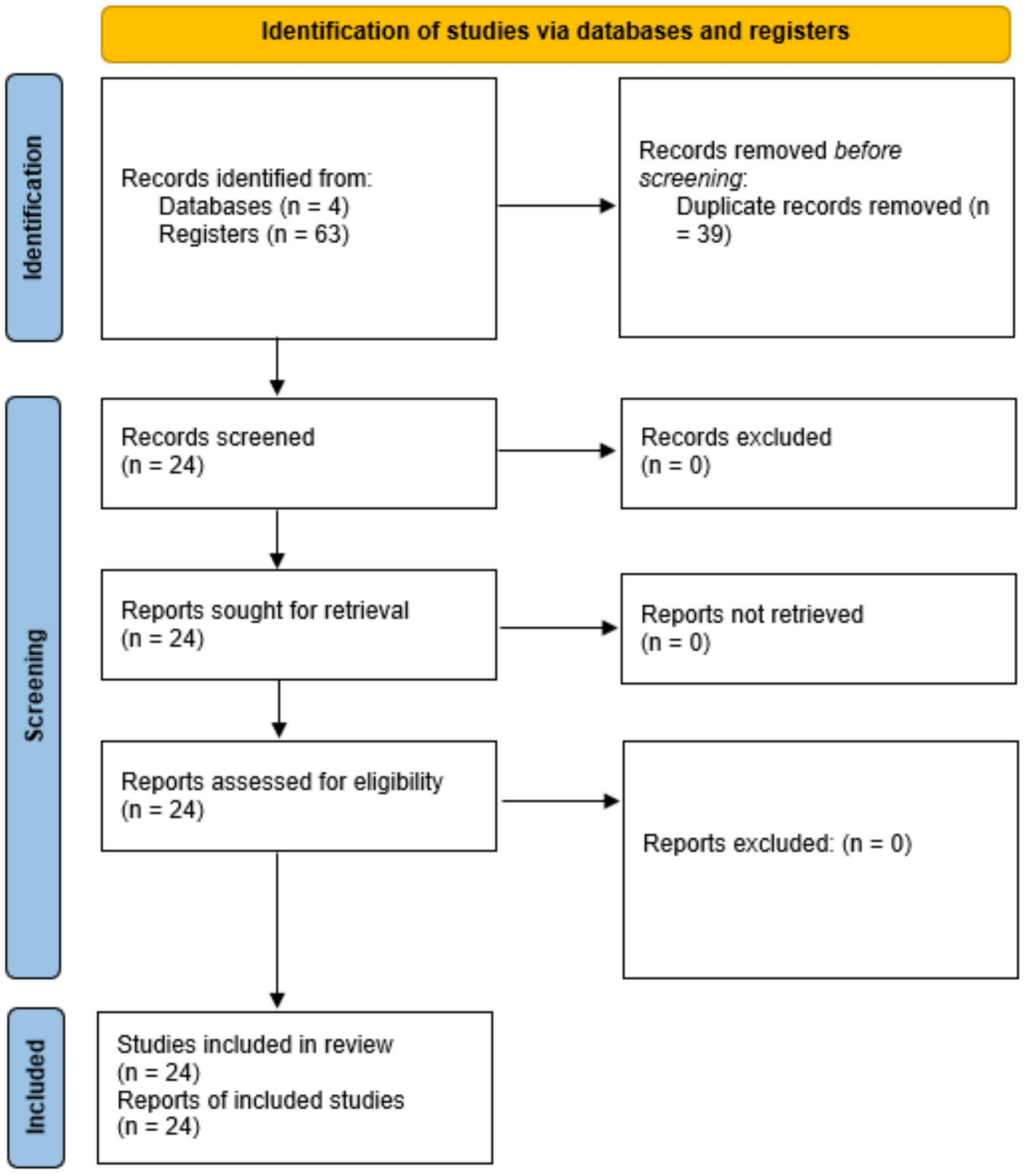
Flow diagram PRIME model use.

Through the research questions, the following search strategies were established in the following databases: WoS, Scopus, PubMed, and SciELO (Table 1). Finally, the information search was updated on 20 November 2024 in the databases indicated above (Figure 1) in the advanced search option.

**Table 1:** Database and record identification by search strategy.

Database	Search strategy	N° of registers
Web of Science	TS = (“Preventable Risk Integrated ModEl”)	19
Scopus	TITLE-ABS-KEY (“Preventable Risk Integrated ModEl”)	22
PubMed	“Preventable Risk Integrated ModEl” [Title/Abstract]	20
SciELO	“Preventable Risk Integrated ModEl” (Todos os índices)	2

Own elaboration.

### 2.4 Selection criteria

The inclusion criteria were: (a) research up to 20 November 2024 that used the PRIME model; (b) development of counterfactual scenarios through PRIME; (c) identification of the word “Preventable Risk Integrated ModEl” in the title and abstract of the research; (d) results associated with risk factors related to diet, tobacco or alcohol consumption, and physical activity; and (e) research in English, Spanish, and Portuguese.

The exclusion criteria were (a) case studies, commentaries, and opinion articles; (b) research that does not refer to the use of PRIME; (c) research available in the databases used; and (d) research that does not specify its use and whose results are not applicable according to the characteristics of the model developed.

## 3 Results

A total of 24 articles were identified for the development of the present review (41, 50–72). The analysis of these publications revealed three main applications of the PRIME model in assessing the impact of dietary and lifestyle modifications on non-communicable diseases (NCDs) (Supplementary Material 2).

### 3.1 Uses of the PRIME model

#### 3.1.1 Scenarios for adherence to dietary policies and health recommendations

Ten studies utilized the PRIME model to develop counterfactual scenarios for predicting population changes in adherence to health-related policies or recommendations.

Scarborough et al. (41), who have established the theoretical foundation of the model, demonstrated that adopting healthy dietary recommendations could reduce up to 33,157 deaths annually in the UK. Building on this foundation, Smed et al. (50) examined taxation effects on saturated fats and found decreased consumption, with a greater reduction in women (4.9%) than in men (1.6%), alongside increased vegetable consumption (7.9%) and fiber intake (3.7%).

Further exploring policy interventions, Labonté et al. (51) determined that 11,715 deaths could be avoided or delayed by reducing the intake of calories, total fat, sodium, and saturated fatty acids through traffic light labeling. Similarly, Pollock et al. (52) estimated that transitioning from a high greenhouse gas emission diet to a low emission diet could reduce or delay 23,739 deaths from CVD and cancer.

The impact of salt reduction policies was specifically addressed in multiple studies. Perera et al. (53) observed that 94,156 deaths from cardiovascular diseases could be avoided by reducing salt consumption by 30% across countries, with this figure rising to 193,155 deaths if WHO salt consumption recommendations were followed. In the Canadian context, Flexner et al. (54) found that reducing sodium intake by 17, 28, and 46% could prevent or delay 2,176, 3,252, and 5,296 CVD deaths, respectively.

Sugar reduction was another focus area, with Flexner et al. (55) reporting that 6,770 deaths from NCDs could be delayed by a 20% reduction in free sugars in foods and beverages. In a more comprehensive approach, Flexner et al. (56) estimated across four scenarios that between 2,183 and 8,907 diet-related NCD deaths could be avoided or delayed through combined reductions in sodium, sugar, saturated fat, and caloric intake.

The effects of food labeling and substitution were examined by Flexner et al. (57), who identified that such scenarios could prevent between 2,148 and 7,047 diet-related NCD deaths, depending on population adherence rates. Complementing these findings, Pourmoradian et al. (58) demonstrated that replacing sugary drinks with water had the greatest impact on reducing type 2 diabetes prevalence compared to other intervention scenarios.

#### 3.1.2 Scenarios for food consumption modification to prevent NCD deaths

Another 10 studies used the PRIME model to predict how changes in food consumption patterns could prevent premature NCD-related deaths, with particular attention to geographical and demographic variations.

Geographic comparisons were highlighted in studies by Alston et al. (59), who identified that 1,461 deaths from cardiovascular disease (CVD) could be delayed or avoided in rural areas, with 1,646 CVD deaths attributable to obesity and smoking. In a subsequent study, Alston et al. (60) expanded this analysis, finding that 40% of CVD deaths could be avoided in both metropolitan (9,673) and rural (5,219) areas by following dietary and lifestyle recommendations.

The effectiveness of multi-nutrient interventions was demonstrated by Goiana-da-Silva et al. (61), who predicted that reducing salt (16%), sugar (20%), and eliminating trans fatty acid consumption would prevent 798 deaths from NCDs, primarily from cardiovascular causes (692). Regulatory approaches were evaluated by Kaur et al. (62), who reported that implementing tighter restrictions through the FSANZ NPSC model was associated with 4,374 fewer deaths per year, mainly from cardiovascular disease (4,078).

Diet quality as a holistic measure was investigated by Julia et al. (63), who demonstrated that groups with better dietary quality had estimated mortality reductions ranging from 1,664 to 3,379 depending on the index used. Focusing specifically on salt consumption, Nilson et al. (64) calculated that 4.001 deaths from cardiovascular diseases could be prevented by 2027 if salt consumption were reduced according to different scenarios.

Multiple risk factor interventions were explored by Breda et al. (65), who estimated that reducing tobacco and salt consumption by 30% and physical inactivity by 20% could avoid 19,859 deaths in 2017, with 85.2% of them from cardiovascular diseases. The dose–response relationship of salt reduction was quantified by Vega-Solano et al. (66), who determined that 295 CVD deaths would be avoided with a 15% salt reduction, increasing to 750 CVD deaths with a 46% salt reduction.

Food substitution effects were examined in detail by Adjibade et al. (67), who found that specific pizza substitutions affected disease risk, with better nutritional options reducing risk by up to 13.9% in frequent consumers while worse options increasing risk by up to 32.4%. Comprehensive health metrics were considered by Burgos et al. (68), who calculated that reducing salt consumption to 5 g/day could prevent 2,656 deaths annually (28.5% of CVD deaths) and prevent 60,529 disability-adjusted life years.

#### 3.1.3 Scenarios related to costs/benefits/expenses in NCD prevention

The economic dimension of NCD prevention was addressed in four studies that developed counterfactual scenarios to predict the expenses, benefits, and costs associated with preventive interventions.

Briggs et al. (69) established a foundational model for estimating the cost-effectiveness of interventions affecting diet and physical activity across multiple disease outcomes, providing a methodological framework for subsequent economic analyses. Building on this approach, Madia et al. (70) quantified potential savings in South Korea, revealing that in an “optimal world” scenario, USD 8.1 billion could be saved, while in a “risk reduction world” scenario, expenses associated with NCDs could be reduced by USD 3.7 billion.

The economic impact on developed Asian economies was assessed by Saito et al. (71), who determined that Japan could have saved USD 35.1 billion in 2019 and avoided 564,000 NCD cases through dietary improvements and tobacco/alcohol interventions. Extending this analysis to Latin America, Espinosa Herrera (72) calculated that Mexico could save USD 3.1 billion in medical care through appropriate consumption of salt, fats, alcohol, fiber, fruits, and vegetables, with an additional USD 342 million saved by transitioning from conventional tobacco to electronic cigarettes or heated tobacco products.

### 3.2 Scientific production using the PRIME model

The bibliometric analysis of the 24 articles revealed significant patterns in the scientific production related to the PRIME model. We established five key analytical dimensions to systematically categorize the findings: (1) author productivity and citation impact, (2) conceptual framework analysis through keyword frequency, (3) international collaboration patterns, (4) dissemination channels and impact metrics, and (5) the temporal evolution of research focus.

**Figure 2:**
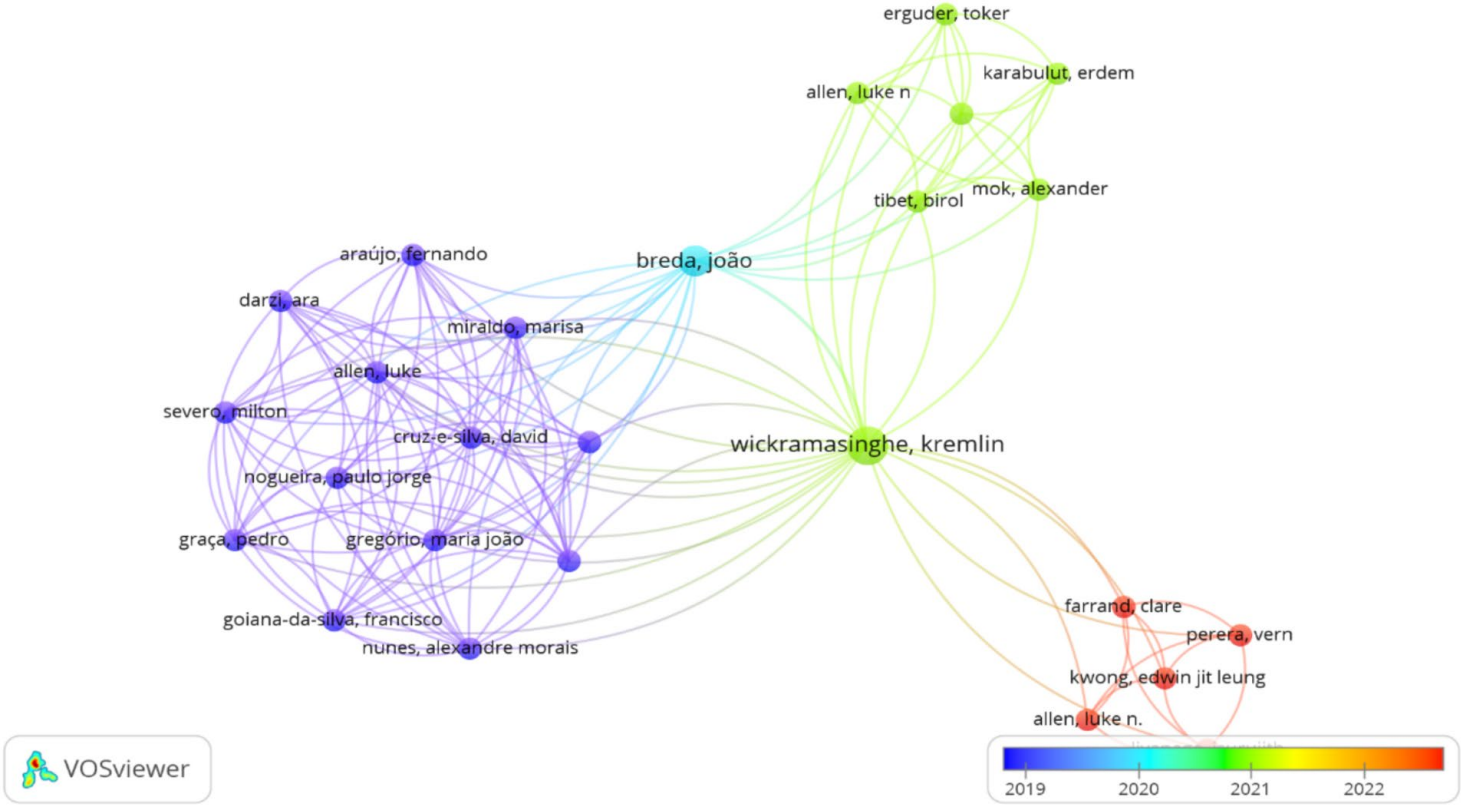
Most cited authors by article number.

The analysis identified a total of 100 researchers contributing to PRIME model applications, with varying levels of productivity and impact. Flexner Nadia emerged as the most prolific author with the highest number of papers (*n* = 4) and 15 citations. In terms of citation impact, Wickramasinghe Kremlin and Breda Joao demonstrated the highest scientific influence with 26 and 25 citations, respectively, despite publishing fewer articles than Flexner. The collaboration network analysis revealed limited connectivity among researchers, with only 26 authors showing direct collaborative connections to other authors in the field (Figure 2), suggesting a fragmented research community with potential for increased collaborative efforts.

**Figure 3:**
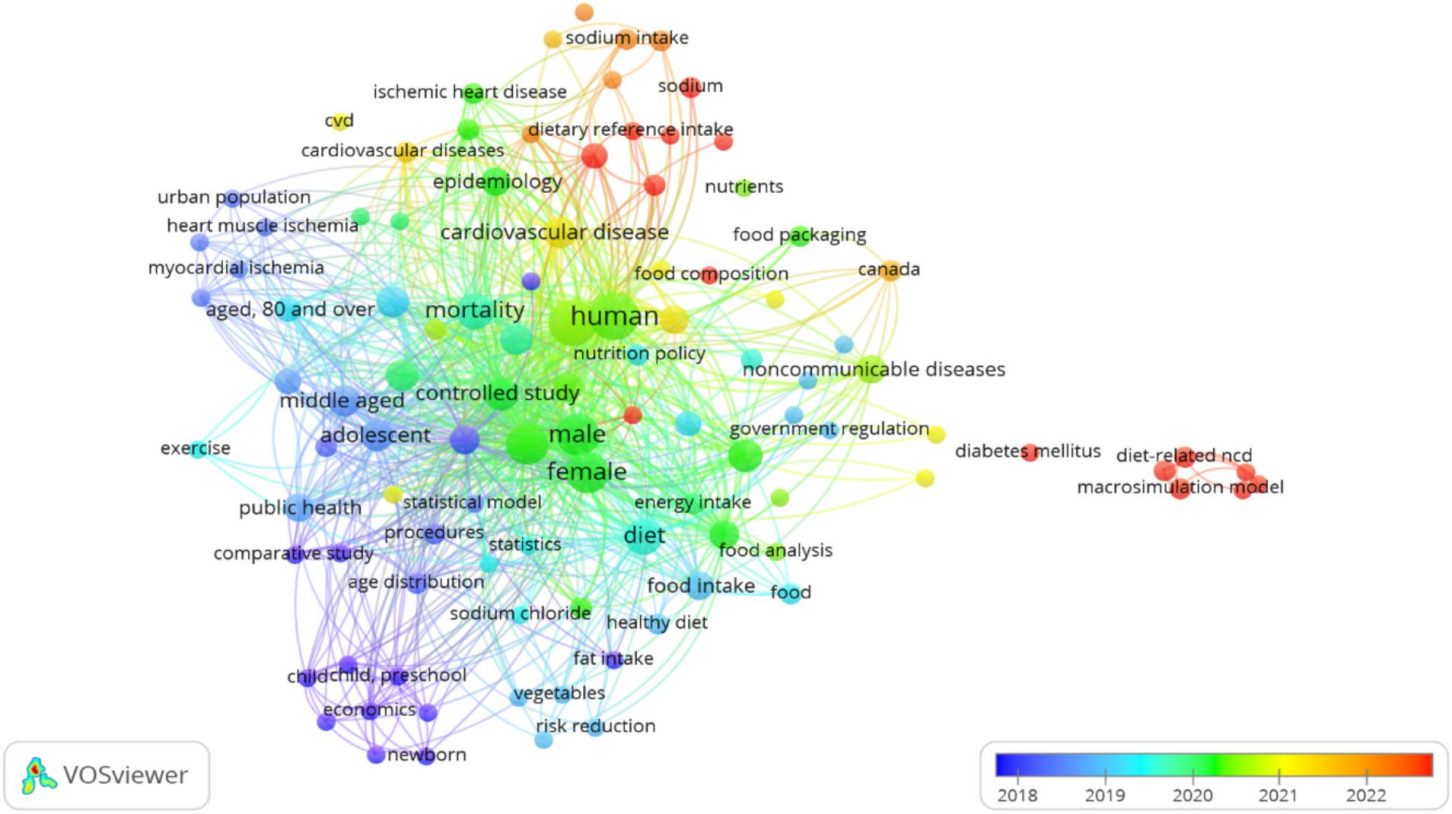
Keywords and concepts most frequently used in the articles.

Keyword analysis identified 247 unique concepts across the 24 articles with 105 concepts occurring at least twice. Six key concepts dominated the conceptual landscape: humans (*n* = 14), adult (*n* = 13), female (*n* = 11), aged (*n* = 10), mortality (*n* = 8), and diet (*n* = 8). This distribution reveals a strong focus on human adult populations with particular attention to female and elderly subjects. The concentration on mortality and diet as primary outcomes and determinants reflects the model’s primary application to mortality prevention through dietary interventions. Notably absent from the high-frequency keywords were terms specifically related to economic assessments or policy implementation suggesting potential gaps in the research focus (Figure 3).

**Figure 4:**
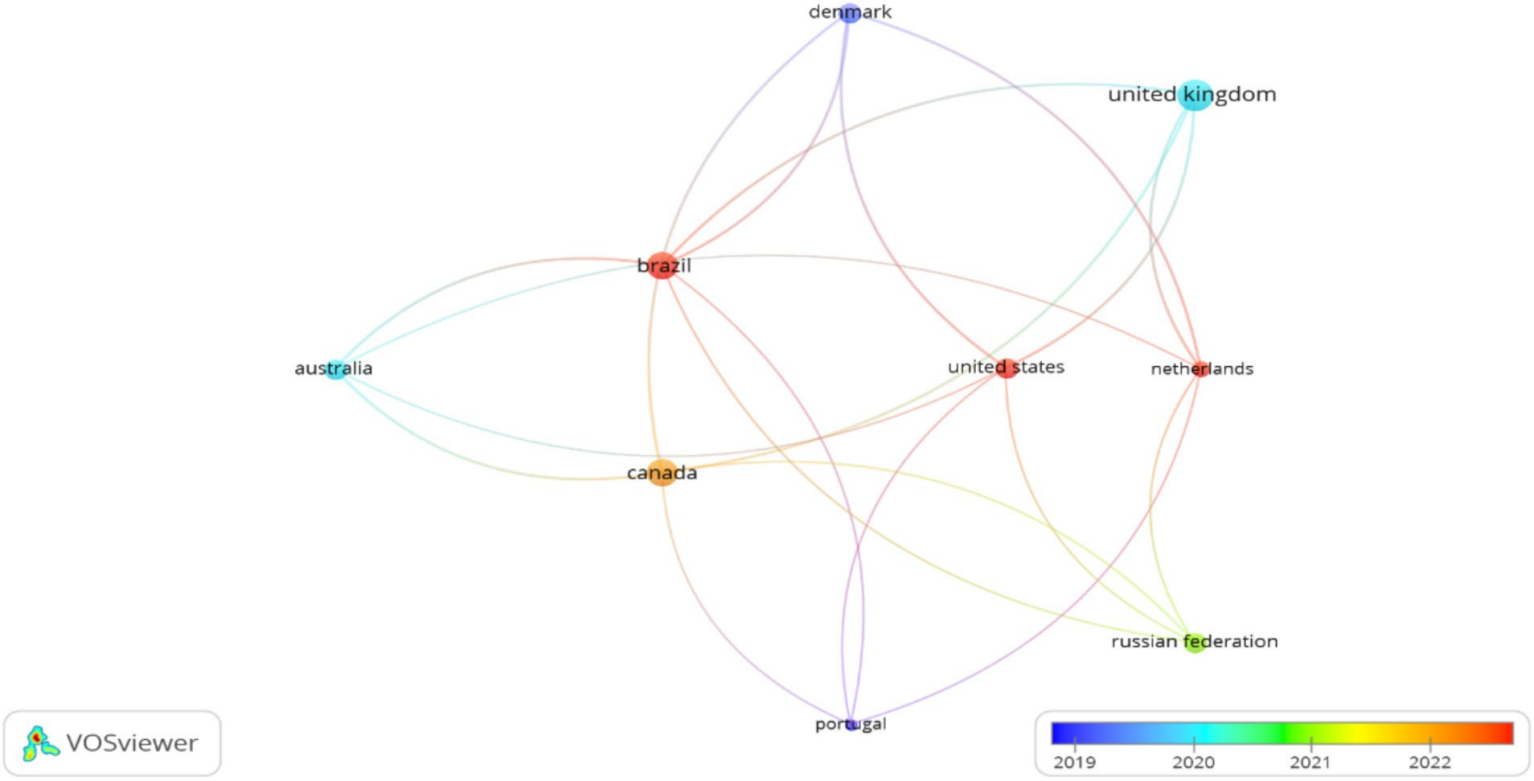
Origin of the countries from which the authors participate.

The geographical analysis identified contributions from 22 countries, with pronounced leadership from specific regions. The United Kingdom demonstrated the highest productivity and impact with 8 documents and 170 citations, establishing its position as the pioneering center for PRIME model research, which aligns with the origin of the model’s development. Four countries showed moderate but significant contributions: Brazil (6 documents, 34 citations), Canada (6 documents, 53 citations), Denmark (3 documents, 107 citations), and Australia (3 documents, 54 citations). The international collaboration network exhibited limited connectivity, with only nine countries (40.9%) showing collaborative connections (Figure 4), suggesting opportunities for expanded international research partnerships, particularly in developing regions where the NCD burden is rapidly increasing.

**Figure 5:**
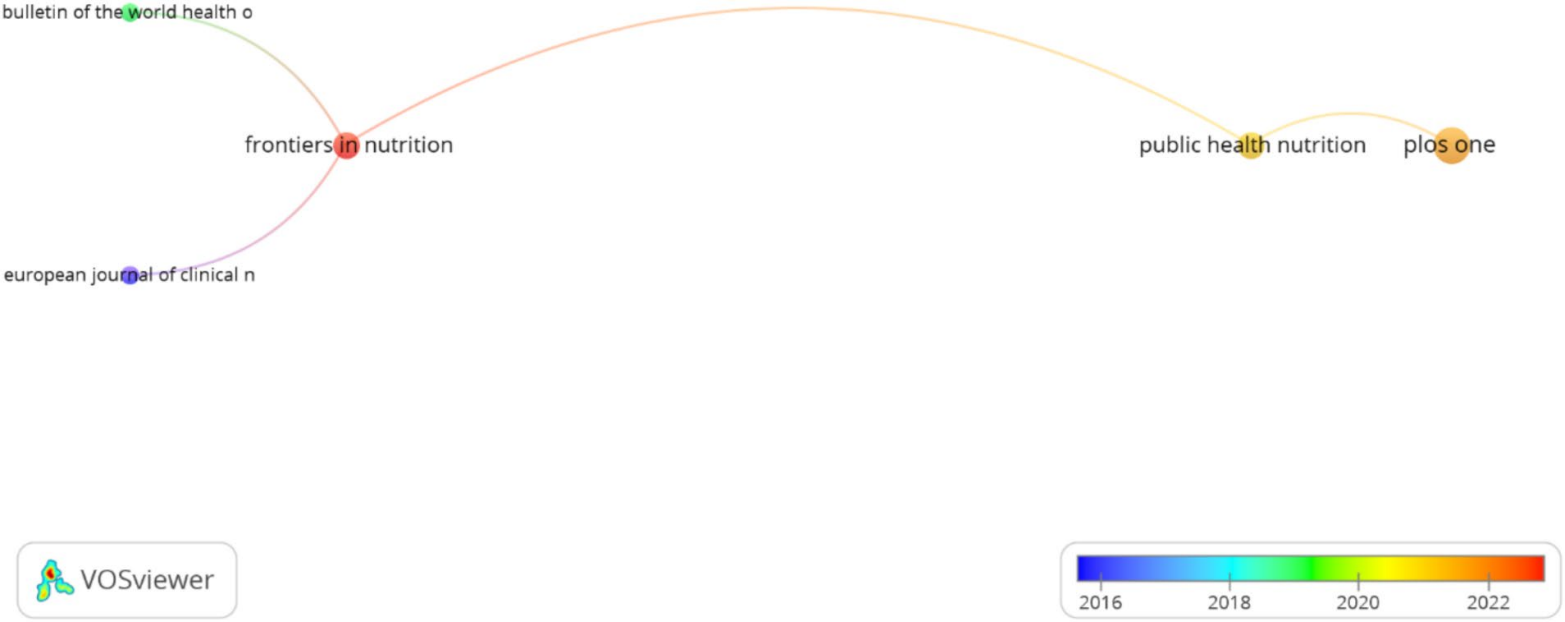
Scientific journals and citations per article published on the subject.

The analysis of publication venues revealed 15 journals publishing PRIME model research, with notable variation in publication volume and citation impact. A strategic pattern emerged where high-impact specialized journals published fewer but highly influential papers: European Journal of Clinical Nutrition (1 paper, 91 citations) and BMJ (1 paper, 31 citations). In contrast, multidisciplinary journals published more papers with moderate citation impact: PLOS One (4 papers, 47 citations) and Public Health Nutrition (2 papers, 26 citations). Limited connectivity among journals (only five showing connections) indicates specialized dissemination channels with minimal cross-disciplinary integration (Figure 5). This pattern suggests that PRIME model research has achieved high impact in specialized nutrition and public health venues but has potential for broader dissemination across health policy and economic journals.

### 3.3 Research focus evolution and methodological approaches

Through chronological analysis of the 24 studies, we identified a progressive evolution in research focus and methodological sophistication. Initial applications (2014–2016) centered on establishing the model’s validity for dietary interventions in high-income countries. Mid-period studies (2017–2019) showed geographical diversification with applications in middle-income countries and methodological expansion to include environmental sustainability impacts. Recent research (2020–2022) demonstrated increased methodological complexity with economic analyses, policy implementation assessments, and applications to specific subpopulations. This evolution reflects the maturation of the PRIME model from a theoretical tool to an applied policy evaluation framework with diversified applications across contexts and outcomes.

## 4 Discussion

This study combined specific methodologies to analyze information from 24 articles that demonstrated the use of the PRIME model and its applications across different contexts. Our findings contribute to a better understanding of this predictive tool for the prevention of non-communicable diseases (NCDs).

### 4.1 Key findings

The PRIME model, established theoretically by Scarborough et al. (41), has emerged as a valuable tool for projecting preventive scenarios related to NCD risk factors. We identified diverse applications, including studies on food consumption patterns (50), sodium intake reduction (53, 54, 64, 66, 68), prevention of NCD-related mortality (51, 52, 55–63, 65, 71, 72), development of new predictive models (69), and food substitution analysis (67). The model’s significance lies in its capacity to project future outcomes that could guide public health policies and potentially reduce healthcare costs, particularly in resource-limited settings (73).

### 4.2 Relationship with existing knowledge

The analyzed studies align with the United Nations sustainable development goals, particularly those related to health (74), and complement previous research on risk factor modification for NCD prevention (3, 10, 40, 75). The geographical distribution of studies across the Americas, Europe, Oceania, and Asia corresponds with epidemiological data showing that 81% of deaths in the Americas are associated with NCDs (76). The global mortality trends observed in 2018 demonstrated that high-income countries in Europe, Oceania, and Asia had lower NCD mortality risks, providing potential benchmarks for interventions in lower-resource countries (77).

The scientific production analysis identified researcher Nadia Flexner (54–57) as a key contributor among 100 authors in the field. The most frequent keywords were “human,” “adult,” “female,” “aged,” “mortality,” and “diet.” Our analysis emphasizes that proper keyword selection is crucial for research visibility and impact (78–82), with appropriate use of Medical Subject Headings (MeSH) being particularly important in health research.

### 4.3 Strengths and limitations

As with all research, our work has limitations that should be acknowledged (83). First, the heterogeneity of research objectives, data sources, and keywords precluded conducting a systematic review or meta-analysis. Second, studies not explicitly mentioning “PRIME” or “Preventable Risk Integrated Model” in their title or abstract may have been missed. Third, the results of individual studies are limited to specific populations or contexts. Finally, the characteristics of the reviewed studies limit the combination of other keywords or the specific use of MeSH terms.

The strengths of our research include demonstrating the application of the PRIME model for projecting the prevention of deaths associated with behavioral risk factors for NCDs. Additionally, we highlighted the collaborative nature of the research using this model across different countries and researchers and its free availability to the scientific community. Finally, our methodological approach allowed visual and narrative analytical presentation of the results.

### 4.4 Implications for practice and policy

Based on our findings, we suggested standardizing keywords for better identification of articles using the PRIME model. Additionally, researchers should provide more detailed methodological descriptions, particularly regarding information from countries outside the study’s origin. The model’s ability to project future health outcomes positions it as a valuable tool for public health policy development and strengthening prevention guidelines for NCDs associated with modifiable risk factors.

### 4.5 Future directions

Future research could focus on developing free software packages for the analysis and visualization of PRIME model data. This would enhance accessibility for non-research sectors, such as public education and health departments that directly interact with populations affected by NCDs. Such tools could facilitate knowledge translation and promote public understanding of how dietary regulation and behavioral changes can modify risk factors and prevent NCDs. Further validation studies across diverse populations would strengthen the model’s applicability in different contexts.

## 5 Conclusion

A total of 24 research studies evidenced the PRIME model, where 22 of them modeled counterfactual scenarios to generate possible changes in a particular population when changes are made in dietary habits, behavioral changes, and adherence to national and international dietary recommendations. These scenarios modeled possible outcomes associated with national or international recommendations related to nutrient consumption, reduction or suppression of tobacco or alcohol consumption, implementation of taxes on certain foods, following nutritional guidelines, implementation of public policies on nutrition, among others, which generated current, ideal, or hopeless scenarios when showing, delaying, or reducing the number of possible deaths or public expenditures associated with the treatment of NCDs or the development of new specific models for risk factors using the PRIME model as a basis. Only two articles were reviewed, one of which established the theoretical basis for the creation of the model and the second associated with the combination of this model with other factors directly related to physical activity.

On the other hand, the author with the greatest contribution in the area was Flexner Nadia. In addition, in terms of keywords, 6 concepts were found to be the most used in the research analyzed. Similarly, out of the 22 countries, the United Kingdom is the country with the greatest contribution, and 5 journals were identified in which this research was published.

Although the PRIME model was created by a group of researchers in collaboration with WHO, to date, there is limited scientific evidence to verify its use more specifically. However, a large group of researchers are using and evidencing the results in the prevention or reduction of deaths and reduction of public expenditure associated with the treatment of NCDs. The results obtained in this research should be considered with caution because each article was developed in a particular population and the quality of the data used.

## Data Availability

The data analyzed in this study is subject to the following licenses/restrictions: contact the corresponding author to request the information. Requests to access these datasets should be directed to Gerson Ferrari, gerson.demoraes@usach.cl.
