# Supplementary material for: Use of Preventable Risk Integrated Model on behavioral risk factors: a scoping review and bibliometric analysis
Source: Front Nutr. 2025 May 9;12:1572234. doi: 10.3389/fnut.2025.1572234 (PMC12100626; doi:10.3389/fnut.2025.1572234)
Supplement: Supplementary file 2 [file Table_2.docx]

Supplementary 2. Purposes, methods and main conclusions of the PRIME model

| Author | Purpose of Research | Methods | Conclusions |
| --- | --- | --- | --- |
| Scarborough et al. [41] | Describe the structure of the PRIME model, and how this tool allows the simulation of scenarios that assess the impact on population health with the reduction of NCD deaths, through a model that estimates possible changes in people's behavioral habits in diet, tobacco consumption, alcohol consumption and physical activity; , the objective was to analyze the results of eleven users of the Preventable Risk Integrated Model (PRIME) tool and previous versions of the model. | Across these four domains, there are twelve behavioral risk factors, and twenty-four health outcomes oriented toward cardiovascular disease, diabetes, cancers, chronic obstructive pulmonary disease, kidney disease, and liver disease. Also, the authors describe the development of a source code for the Python PRIME program which can be analyzed and a web application for its use that would be available from 2015. | The use of PRIME allows the modeling of possible scenarios to estimate changes in behavioral habits related to diet, physical activity, tobacco and alcohol consumption, thus, it is possible to reduce costs associated with the management of diseases associated with these habits. |
| Smed et al. [50] | Assess the effect of the Danish saturated fat tax in terms of changes in dietary nutritional quality, i.e., changes in saturated fat consumption, as well as other non-specific dietary measures, and to model associated changes in mortality for different age groups and genders. | For the development of the research, information on about 2,500 different families from 2009 to 2012 was obtained from the Scandinavian Panel Services (GfK) related to consumers, for information related to food nutrients we used data from the Danish Food Composition Data Bank (available from the National Food Institute from their website, in case the information for some foods is missing we consulted the suppliers' website) related to fats, carbohydrates, sugar, sodium and calcium in foods. To estimate the effects of the consumption tax, a parametric model of changes in nutrient intake was developed taking as reference age, sex and marital status, among other in-formation related to consumption and purchases. In addition, the PRIME model was used to develop the estimates. | The application of the diet quality tax allowed a decrease in the consumption of saturated fats. In addition, this increase allowed an increase in the consumption of fruits, vegetables and fiber. It was also possible to estimate a reduction in deaths from NCDs; however, these changes also had the opposite effect of increasing the consumption of some foods that were harmful to health. |
| Labonté et al. [51] | Model the potential impact of traffic light labeling (TLL) adoption on noncommunicable disease (NCD) mortality in Canada, due to the previously predicted improvement in nutrient intake. | For the development of the research, data available from 19,915 participants from the Canadian Community Health Survey (CCHS) cycle 2.2 (2004), related to the 24-hour dietary recall, were used. Based on information available from a previous study, red, amber, or green color codes were assigned for nutrients, which were total fat, saturated fatty acids, sodium, and total sugars. The color distribution was based on the 2013 UK Front-of-Package (FoP) Nutrition Label Guidance. Foods with at least one red traffic light were replaced, where possible, with healthier, non-red colored products, e.g., lean meat was replaced with extra lean meat, and so on as appropriate. In the case of not having a change food, this food remained on the list. A dietitian was involved to identify foods from the Canadian Nutrient File 2001b, data from the University of Toronto Food Label Information Program 2010 and Food Label Information Program (FLIP) 2010. | If Canada adopts the measure of labeling food through traffic lights, deaths associated with NCDs could be reduced. |
| Pollock et al. [52] | Verify whether American diets with the highest carbon footprints predict higher mortality at the population level due to diet-related chronic diseases than those with the lowest carbon footprint. | To obtain the information, the PRIME model was used to simulate annual cardiovascular and cancer deaths that could be avoided by changing to foods with lower greenhouse gas (GHG) emissions. Data from 12,865 subjects aged 18 to 64 years from the National Health and Examination Survey (NHANES) 2005-2010 related to the 24-hour dietary record were also used, in addition to the use of a database on the impact of the diet linked to the environment (DataFIELD). Subsequently, the GHG of the foods mentioned in NHANES 2005-2010 were calculated, grouping them by quintiles, age group and sex, and then classifying them into a model of diets with a higher carbon footprint (HiGHGE) and lower (LoGHGE). For information related to mortality, ICD-9 was used as a reference. | Carrying out this simulation using the PRIME model allowed them to demonstrate that a diet with HiGHGE is responsible for a greater number of deaths from CVD and cancer, which could be avoided by changing this behavior towards LoGHGE diets. |
| Perera et al. [53] | Estimate the number of deaths that would have been avoided if the salt intake levels of the relevant countries had been reduced by 30% to achieve SDG 3.4. | For the development of the re-search, data from the STEPS 2016 study were used for Armenia and Belarus. To obtain data for Kazakhstan, information from two cross-sectional surveys related to sodium and potassium consumption was used, using 24-hour urine samples. For Kyrgyzstan, WHO reference data were used regarding the availability of sodium in foods (this country does not have such information). For the Russian Federation, information on salt consumption available in the 2018 survey on the dietary structure of its population was used. For the analysis of the available information, the PRIME model was used to estimate the number of preventable deaths through hypothetical changes in NCD risk factors; salt intake was used for this study. Subsequently, a scenario with a 30% reduction in salt consumption for the country and another scenario considering the adoption of WHO recommendations (5 g/day of salt) were developed. Then, population and NCD mortality data were taken from each country. | The reduction of salt consumption by the inhabitants of the countries in the study developed could allow a reduction in deaths from NCDs by one third, to achieve SDG 3.4. |
| Flexner et al. [54] | Obtain robust estimates of Canadians’ usual sodium intake by linking the 2017 Food Label In-formation Program (FLIP), a database of Canadian branded foods and beverages, with food intake reported in the 2015 Canadian Community Health Survey (CCHS)–Nutrition; model sodium intake if foods had been reformulated to align with Health Canada’s sodium reduction reformulation targets for processed foods; estimate the number of CVD deaths that could be avoided or delayed if Canadian adults reduced their average sodium intake to recommended levels under three scenarios, the first scenario was 2,300 mg/d, driven by a systematic reduction of sodium levels in packaged foods (reformulation); the second scenario was 2,000 mg/d, to meet the World Health Organization (WHO) recommendation; and the third scenario was 1 | To meet the research objectives, the researchers performed the following actions: information was obtained from the Canadian Community Health Survey – Nutrition (CCHS-Nutrition 2015) to estimate the sodium intake of 20,176 participants and a total of 13,612 adults for the specific objectives. In addition, the Food Label In-formation Program (FLIP) database was used, where it was subsequently merged with the Food and Ingredient Details (FID) file used in the CCHS-Nutrition 24-hour recall. Subsequently, the scenarios were created, estimating the specific sodium intake by age and sex, considering the information available in FLIP 2017, FID and CCHS-Nutrition. Regarding the creation of the first scenario for reformulation, the information available in FLIP and CCHS-Nutrition was considered, considering the reference information in the Health Canada Guide for the food industry on the reduction of sodium in processed foods considering the levels of adequate intake and Intake for the Reduction of Chronic Diseases. For the creation of the second (WHO 2,000 mg/d) and third (adequate intake of 1,500 mg/d) counterfactual scenarios, compliance with the recommendations indicated above was evaluated, compared to the average intake of the study population. Although the recommendations are oriented at an individual level, these analyses were carried out at the population level, because some people consumed less sodium than recommended. Regarding the information regarding the demographics and burden of CVD mortality in the population, it was obtained from the CANSIM tables of Statistics Canada, and the ICD-10 was used for mortality data. Finally, for the health impact modeling, the PRIME model was used to estimate the number of CVD deaths that could be avoided or delayed for this study. | If the canadian population were to reduce sodium intake in line with current recommendations, lives could be saved by preventing or delaying CVD deaths associated with sodium consumption in foods or beverages. |
| Flexner et al. [55] | Estimate the number of diet-related NCD deaths that could be avoided or delayed if Canadian adults reduced their caloric intake as a result of a systematic 20% reduction in the free sugar content in foods and beverages in Canada. | For the development of the research, reference and counterfactual scenarios were created using the PRIME model. To obtain in-formation related to diet, the Canadian Community Health Survey - Nutrition (CCHS Nutrition 2015) was used from a total of 20,176 people. This survey has a 24-hour dietary recall (to estimate caloric intake), in addition to data from recent research conducted by the research group, developing a scenario for the systematic 20% reduction of free sugars in foods and beverages. The Food Labelling Information Program (FLIP) 2017 database was also used to estimate free sugar and calorie levels in foods and beverages. For population information, a 2019 demographic study on deaths associated with CVD, cancers, diabetes, chronic kidney and liver failure was used, obtained from Statistics Canada's Canadian Socio-economic Information (CANSIM), and deaths were based on the ICD-10. | Considering this created scenario but only reducing the consumption of sugars in food and drinks to 3.2%, could delay or avoid 7.5% of total deaths from NCDs. |
| Flexner et al. [56] | Estimate the potential dietary impact among Canadian adults of the implementation of a mandatory front-of-package label with high "ingredient" content based on real experimental and observational evidence; estimate the number of diet-related NCD deaths that could be avoided or delayed due to these estimated dietary changes using simulation modeling methods. | For the development of the research, the reference data from the Canadian Community Health Survey - Nutrition 2015 (CCHS-Nutrition) were used, where the 24-hour recall information from the CCHS-Nutrition 2015 Public Use Microdata File (PUMF) was used. In addition, the Canadian Nutrient File (CNF) from Health Canada - 2015 was used for the inclusion of data from 11,992 participants. Once the information was obtained, four counterfactual scenarios were created, the first of them was composed of the early evaluations of the Chilean Food Labelling and Marketing Law, the second scenario was composed of the Chilean Food Labelling and Marketing Law – disaggregated changes by food and beverages, the third scenario was composed of a randomized experimental study of the Canadian market: disaggregated changes by snacks and beverages, and the fourth scenario was composed of a meta-analysis on the impact of nutritional warning labelling: general changes. In the four scenarios, the nutritional content related to the consumption of sodium, sugars, saturated fats and calories were considered for the analyses. Finally, the information related to demographics and mortality due to di-et-related NCDs in Canada was obtained from CANSIM (Statistics Canada), CCHS-Nutrition 2015 and 2019, and for the data they were obtained from the ICD-10. | The implementing front-of-package “High in Fat” labeling could reduce sodium, sugar, and saturated fat intake, potentially preventing or delaying diet-related deaths from NCDs in 8,907 Canadians. |
| Flexner et al. [57] | Estimate the potential dietary impact of replacing packaged foods actually consumed by Canadians with similar packaged foods that displayed at least one "high in" symbol less; estimate the number of deaths from diet-related non-communicable diseases (NCDs) that could be avoided or delayed due to the estimated dietary impact of food substitution. | For the development of the research, reference dietary data obtained from the CCHS-Nutrition 2015 from a total of 11,992 subjects were used. In addition, from this same survey, the data on average height and BMI by age and sex groups of the reference food intake were used. Also, data available from the CCHS-Nutrition 2015 food and individual ingredient details file were used, as well as information available from Health Canada's 2015 CNF. Likewise, the Food Label Information and Pricing Database (FLIP) was used. Further-more, products available in FID and FLIP 2017 were categorized according to Health Canada's Reference Quantity Table (TRA), being evaluated according to FOPL regulations. Regarding the creation of counterfactual scenarios (1st scenario of 30%, 2nd scenario of 50%, 3rd scenario of 70% and 4th scenario of 100%), they were based on data from the "High in" regulations of Chile and Israel. Finally, the PRIME model was used for the health impact model. For the NCD policy scenarios, the number of inhabitants of Canada in 2019, the annual number of diet-related NCD deaths, reference estimates of the intake of energy, sodium, saturated fat and fiber in the Canadian population, and counterfactual estimates of the intake of energy, sodium, saturated fat and fiber in the Canadian population after modelling counterfactual scenarios for food substitution were included. In this way, the available information from CANSIM Statistics Canada, related to demographics, diet-related mortality, and for the diet-related mortality data, the ICD-10 was used. | The canadians could improve their intake of healthier nutrients if foods were labeled “High in.” Thus, reducing sodium and total sugars could delay or prevent deaths from food-related NCDs. |
| Pourmoradian et al. [58] | Quantify the differences in the prevalence of type 2 diabetes, and to compare the number of deaths from type 2 diabetes that could be avoided between the implementation of various nutritional policy options to reduce the consumption of sugary drinks and the non-implementation of policies from 2015 to 2035 in the Iranian population. | For the development of the research, the PRIME model was used, in addition, the following epidemiological information was obtained from Iran through the AZAR 2015 cohort study, in addition, the available information from the Food Frequency Questionnaire (FFQ) was used to obtain the quantity and frequency of food and beverages. On the other hand, and considering previous epidemiological studies, the direct and indirect effects of SSBs consumption on DM were modeled. Regarding the information on the incidence and mortality rate of type 2 diabetes in Iran, it was based on the available data from the National and Subnational Burden of Diseases, Injuries and Risk Factors (NASBOD) in Iran for 2015, as well as a study conducted by Derakhshan et al. (2014). Finally, for the creation of the simulations, PRIME was used, a scenario was developed to predict the impact of reducing sugary drink consumption on incidence, mortality and the number of deaths postponed or prevented (DPP) from type 2 diabetes over 20 years (2015-2035) under various policy scenarios. The application of a 10% tax on beverages, a 30% reduction in sugar content in sugary drinks, and a “no policy” scenario were considered. | By simulating the impact of nutritional policy in Iranians with type 2 diabetes, they could allow to take preventive measures in the application of policies and to anticipate the effectiveness in preventing or reducing deaths associated with nutrition. |
| Alston et al. [59] | Quantify the differences in the prevalence of major CVD risk factors between rural and metropolitan populations, and to determine the number and proportion of rural deaths from CVD, and specifically from ischemic heart disease, that could be avoided or delayed in rural areas if the levels of risk factors were equivalent to those in metropolitan Australia. | For the development of the research, the PRIME model was used, population information (metropolitan or rural) was obtained from the 2011 Australian Census, as for risk factors were obtained from the 2011-2012 Australian Health Survey (AHS), where average dietary intake, tobacco and alcohol consumption, physical activity level and BMI were obtained. Both sources are accessible through the ABS TableBuilder online program. Mortality data was obtained from the Australian Institute of Health and Welfare (AIHW) providing information by CVD and IHD (considering the ICD-10 classification) in 2011. | There is the potential to reduce or delay CVD deaths in rural Australians if risk factors such as obesity and smoking are reduced in the population. |
| Alston et al. [60] | Determine how many CVD and IHD deaths would be delayed or avoided if Australians complied with public health recommendations in both metropolitan and rural areas; determine which recommendations would be the highest priority in metropolitan and rural populations, based on potential mortality reductions; determine the extent to which absolute and relative ine-qualities in CVD and IHD mortality between rural and metropolitan Australia would persist if public health recommendations were met. | For the development of the counterfactual scenarios and thus the fulfillment of the objectives, the PRIME model was used for the creation of a “Metropolitan” model and the “Rural” model was created, in both models separately were created to determine deaths from CVD and CID to see if they met the recommendations on diet, consumption of tobacco, alcohol and physical activity. For data related to age, sex, area of residence (metropolitan or rural) was extracted from the 2011 Australian CENSUS. As for information on modifiable risk factors, it was obtained from the 2011-2012 National Nutrition and Physical Activity Survey (NNPAS), a component of the Australian Health Survey (AHS), and for obtaining in-formation related to tobacco, alcohol and physical activity consumption, the TableBuilder platform of the Australian Bureau of Statistics was used. Diet data were obtained from Microdata of the Australian Bureau of Statistics. Finally, for mortality data, the ICD-10 classification available in the National Mortality Database of the Australian Institute of Health and Welfare was used, while data from the Cause of Death Unit Record File are provided to the Australian Institute of Health and Welfare by the Registries of Births, Deaths and Marriages and the National Forensic Information System. In addition, a second counterfactual scenario was performed considering that the population never smoked. | If the “Metropolitan” and “Rural” areas of Australia followed the recommendations on smoking, alcohol, diet and physical activity, CVD and IHD deaths could be reduced. Being the “Rural” area the most affected in fat intake. |
| Goiana-da-Silva et al. [61] | Model the reduction of premature deaths attributed to noncommunicable diseases if the processed food reformulation targets agreed between the Portuguese Ministry of Health and the food industry were met. | For the fulfillment of the objective, we used data on dietary intake, mortality from noncommunicable diseases and demographic data from 2016 (for the projection of deaths avoided), we also used data available in the population aged 15-84 years from the National Survey of Food, Nutrition and Physical Activity of Por-tugal 2015-2016, where we obtained the information corresponding to food consumption evaluated per 24 hours. On the other hand, data were obtained for the population of Portugal from the years 1990 to 2016, which were provided by the Portuguese General Directorate of Health. Similarly, for obtaining information related to NCDs, ICD-10 was used, classifying diseases into diseases of the circulatory system, diabetes, malignant neoplasms and chronic respiratory diseases. In addition, a modeling of NCD death trends between 1990 and 2030 was developed, and the possible impact of food refor-mulation on mortality trends, which was performed through the PRIME model. For the information analysis, the data corresponding to food consumption and its transformation into total energy, sodium and fat, the software Electronic Assessment Tool for 24-hour recall (eAT24) was used. For the assessment of dietary exposure, the Statistical Program for Assessing Dietary Exposure software was used, belonging to the Dutch National Institute of Public Health and Environment, Bilthoven, The Netherlands, in addition, consumption data from the National Survey 2015-2026 were combined. And as for the development of counterfactual scenarios for the reduction of premature deaths due to NCDs, the PRIME model was used. | According to the results obtained, it is possible to prevent deaths from NCDs by considering changes in risk factors, considering the regulation of the food industry for the acquisition of improved dietary patterns in Portugal. |
| Kaur et al. [62] | Research questions, which were What would be the impact on NCD mortality rates in the UK if a nutrient profiling model was used to support HRC legislation, so that only foods that passed a nutrient profiling model were eligible to carry HRCs, and What would be the impact if foods that contain HRCs but do not pass the nutrient profiling model were reformulated to meet the model criteria? For the development of the research, the PRIME model was used, incorporating data from the “Role of health-related CLaims and sYMBOLs in consumer behavior” project. | To obtain the information, data from the Living Costs and Food Survey (LCF) 2015 and the National Diet and Nutrition Survey (NDNS 2008-2012) of the United Kingdom were used, where population nutrient intake data, food sales data in grams or milliliters per food group were used. In addition, nutrient composition data from the UK Nutrient Data Bank were used. As for population data by age and sex they were obtained from the 2013 UK Annual Mid-Year Estimates, and mortality, they were extracted from deaths recorded in England, Wales, Scotland and Ireland. For scenario development, parameters from a previously conducted study related to health-related claims (HRCs) were used considering data from the UK food-based Eatwell Guide. Subsequently, two scenarios “restricted HRCs” (1a, 2a) and “reformulation of foods to contain HRCs” (1b, 2b) were created. As for model 1a used the nutrient profile passing the FSANZ NPSC test and model 2a the one proposed by the EU. As for the “reformulation” models was restricted with a nutrient profile model, maintaining the prevalence of HRCs, but would be removed because they do not contain the average nutritional quality of foods that do not contain HRCs. | Limiting the use of CRH could have favorable or unfavorable health benefits, which depends on the manufacturers in the use of this nutrient profiling model. |
| Julia et al. [63] | Compare the magnitude of estimated avoided deaths from chronic diseases obtained by improving the quality of the diet in the French population, measured by a variety of dietary indices. | For the development of the study, a sample of 99,641 individuals with at least three dietary records during the first two years of follow-up of the NutriNetSanté cohort study conducted in 2009 was used. For the development of the study, dietary data related to more than 3,000 foods were used, reporting the consumption of all foods and beverages on two non-consecutive days and a weekend for a period of 2 weeks. As for the calculation of the dietary indexes, they were made according to the nutritional and food recommendations of France (PNNS-GS), the PANDiet diet and a dietary index of US guidelines. On the other hand, the DQI-I and the HDI were used to verify adherence to the Medi-terranean diet. As for the calculation of the profile of the foods consumed, they were based on the FSAm/HCSP DI, in this way, the foods were classified as lower quality, medium quality or higher quality. For sociodemographic data and lifestyles, data from the French national statistical office INSEE were used, for tobacco consumption, self-reported data from the NutriNetSanté cohort study (2009) were used, and the French version of the IPAQ short version was used to obtain PA-related data. Regarding the results, the researchers pointed out that those with lower income, younger age, lower income, higher educational level, tobacco use, and lower PA were mainly men. On the other hand, the higher the nutritional quality of the food, the lower the intake of energy, fat, saturated fat and cholesterol. Regarding the results obtained in the use of the PRIME model, he noted that the intake of low-quality food is associated with a higher number of deaths, compared to those people who modify their diet from medium quality to very high quality is associated with a reduction in a number of deaths. Finally, the researchers pointed out that the results obtained in the research allow highlighting the importance of a healthy diet, specifically, the consumption of a very high-quality diet prevents deaths associated with poor nutrition and consequently the development of NCDs. The dietary data analyses presented reveal significant differences in nutrient intake and dietary patterns according to the quintiles evaluated, highlighting consistent associations between diet quality and nutritional indicators | A stricter regulations and product reformulation could generate important public health benefits, but the observed uncertainty highlights the need for future research to improve model accuracy and validate observed effects. These results provide a solid basis for guiding public policies to optimize nutrition labeling and reformulation strategies to reduce the burden of chronic disease. |
| Nilson et al. [64] | Evaluate the impact of different policy scenarios for the reduction of sodium consumption, based on processed and ultra-processed foods, on the prevention of deaths from cardiovascular outcomes in the Brazilian adult population | To obtain the data, the following secondary information sources were used, for the corresponding information on sodium levels of processed and ultra-processed foods the POF 2008-2009 (Pesquisa de Orçamentos Familiares 2008-2009) was used, to obtain the sodium consumption of the Brazilian population in 2013 the information available from laboratory analysis of the Brazilian National Health Survey (PNS, 2013), for information related to demographics and mortality, information available from the Brazilian Institute of Geography and Statistics (IBGE) and the Brazilian Mortality Information System of the Unified Health System (SIM/SUS) was used, and finally, causes of deaths were considered from the International Classification of Diseases 10th revision (ICD-10). For the information analyses, the 2017 sodium consumption estimate was used as opposed to the 2013 sodium consumption, and the changes in sodium consumption were calculated from the information obtained in the 2008-2009 POF. In addition, the baseline sodium consumption in 2011 and the counter-factual scenarios in 2017 were considered for the development of scenarios from the most unfortunate to possible scenarios considering the Brazilian legal regulations, taking into account the recommendations of the WHO. In addition, also using PRIME, estimates of annual deaths from NCDs by sex and age group were included, considering behavioral risk factors according to the counterfactual scenario of interest related to salt consumption. | The use of the PRIME tool allowed them to create scenarios considering the long-term reduction in salt consumption and the possibility of delaying or decreasing the number of deaths from NCDs if the necessary measures created in the scenarios are adapted. In this way, the developed macrosimulation of mortality could allow the development of improved strategies from public policy related to actions for the development of evidence-based clinical training and education programming for the reduction of the burden of disease, nutritional and food training and education, and cost-benefit economic evaluation for the development of policies mentioned above |
| Breda et al. [65] | Estimate the number of deaths that would be avoided if Turkey fully met its tobacco, alcohol, salt and physical inactivity targets. | They also set out to quantify the relative contribution of each risk factor and model the impact associated with setting different risk factor reduction thresholds: 10%, 20% and 30%. Understanding the relative contribution of different risk factors and thresholds can help inform the prioritization of NCD policy options as Turkey strives to meet its SDG commitment to reduce premature deaths from NCDs by one third by 2030; model the number of deaths that would be avoided if the population complied with the Turkey-WHO recommended limit of 5 g of salt per person per day; to model the impact of increasing the proportion of the population consuming at least one serving of fruits and vegetables by 10%, 20% and 30%. To obtain the results, the re-searchers used the PRIME scenario modelling tool, then used four sources of age-related information: NCD mortality data, distribution of risk factors by sex and age in 5-year time frames and data on the counterfactual distribution of behavioral risk factors. For this, the available data on the population structure of Turkey in 2017, mortality statistics from the Turkish National Institute of Statistics, the 2017 'STEPwise approach to Sur-veillance' (STEPS) survey which was developed jointly with the Turkish Ministry of Health and WHO Europe, the 2010 Turkish Nutrition and Health Survey (TNHS) and the 2012 SALTURK II study which assessed 24-hour urinary sodium levels were used. Thus, and for the development of the model, data on tobacco consumption (STEP 2017), alcohol (STEP 2017 and TNHS 2010), physical inactivity (STEPS 2017), salt (STPS 2017, THNS 2010 and SALTURK II 2012), finally for fruit and vegetable consumption (THNS 2010 and WHO recommendations) were obtained. Subsequently, four scenarios were created, first the Main Analysis was performed: achievement of the objectives of the multisectoral action plan, this scenario is related to the number of deaths observed if the objectives of the action plan had been achieved. A 30% reduction in salt intake was modeled, a 30% reduction in smokers, a 10% reduction in the sedentary population, and the number of deaths associated with the change in these risk factors and in general were modeled; then a Sensitivity Analysis was performed: alternative reference data for salt intake, two sensitivity analyses were performed using alternative data for salt in-take, the first was using the data from SALTURK II and the second reducing the reference levels of STEP (by 25%) and using the standard deviations of THNS. Subsequently, an Estimation of the impact of different levels of policy intervention was carried out, each risk factor was considered and the impact associated with different objectives was modelled, carrying out three Monte Carlo simulations for each risk factor, considering more fruit, vegetable, salt and alcohol consumption, considering relative reductions of 10%, 20% and 30% of the demographic band. Finally, the four contractual scenarios were Objectives of the Multisectoral Action Plan, Sensitivity analysis: objectives of the Multisectoral Action Plan with alternative data on salt, Estimation of the impact of different levels of policy intervention and Comply with the recommended limit of 5 g/day of salt. | According to the modeling carried out by the PRIME model, 20,000 deaths would have been avoided in 2017, thus, the reduction of salt and physical inactivity as main behavioral risk factors could avoid that approximate number of deaths in the Turkish population. |
| Vega-Solano et al. [66] | Estimate the impact of reducing salt intake on CVD mortality in Costa Rica using a scenario simulation model established in 2018. | For the development of this objective, the PRIME model was used to estimate the number of deaths that could be avoided or delayed due to CVD in the population of Costa Rica (considering the country's recommendations, the WHO recommendations on reducing salt consumption), data available from the Household Budget Survey (EPH) of 2012-2013 were used. On the other hand, counterfactual scenarios were estimated with a caloric intake of 2,000 kcal/day (9.0 ± 5.0 g/day) and 2,171 kcal/day (10.0 ± 5.4 g/day), and the information available in the National Nutrition Survey (ENN) 2009 was used. Subsequently, two counterfactual scenarios were created, the first one with a 46% reduction in salt consumption for an energy intake of 2,171 kcal (scenario A) and the second one with a 15% reduction in salt consumption for an energy intake of 2,171 kcal (scenario B). As for scenario A, it was based on the recommendations “National Plan for Reducing Salt/Sodium Consumption in Costa Rica, 2011-2021” and as for scenario B, it was based on the “National strategy for the com-prehensive approach to non-communicable diseases and obesity”. To obtain demo-graphic and CVD mortality information, the information available from the National Institute of Statistics and Censuses (INEC) of Costa Rica for 2018 was used, and for the causes of death the WHO ICD-11 classification was used. A total of 5,649 CVD deaths were considered for the analysis in 2018. | The reduction of salt consumption by the population of Costa Rica could prevent or delay the number of deaths from CVD, because the information obtained through the PRIME model allowed to estimate deaths if salt consumption is reduced. |
| Adjibade et al. [67] | Evaluate the potential impact of pizza substitutions on the risk of type 2 diabetes compared to substitutions with foods from the different categories of combination dishes and to identify the characteristics of pizzas related to a stronger reduction in the risk of type 2 diabetes; examine whether the impact of personalized substitutions, that is, the implementation of a substitution based on an individual dietary risk assessment, differed from that of generic substitution, that is, the same substitution for all individuals. | For the development of the research, the following information was obtained from the participants from the 2nd Individual and National Survey on Food Consumption (INCA 2) 2006-2007, which was developed by the French Agency for Food, Environmental and Labor Safety, with prior authorization from the French National Commission for Information Technology and Individual Liberties. In-formation related to demographics, socioeconomic status, physical activity, food intake, and beverages was used. On the other hand, information from 353 pizza recipes was used to estimate their nutritional composition and proportions, considering ingredients of animal and vegetable origin, type of dough, among other information available from the Food Quality Observatory (Oqali). In addition, the risk change rate for D2 was estimated using the PRIME model. Similarly, analyses were performed to identify the best or worst pizzas for each participant. Subsequently, three types of pizza substitutions were made for those who consumed it, which were considered the 5 best or worst pizzas and an individualized change was made. As for the second substitution, the pizzas with the highest frequency (better or worse), a generic change was made, and the third substitution considered the 78 possible mixed foods from INCA 2 for a generic change. As for the results, the pizza consumers were mostly men, single, smokers, with low consumption of fruits and vegetables, compared to men and women who do not eat them. As for the best pizzas, four groups of them were identified, where they contained a high proportion of vegetables, only one of the four groups had a non-whole grain crust. On the other hand, as for the worst pizzas, a low proportion of vegetables and a high pro-portion of processed meat were found. As for the rates of change for D2 risk, the rates of change were lower in women, in addition, this action allowed to reduce the risk of this NCD in the entire population considering the substitution of generic and personalized pizzas. As for the change of one of the worst pizzas (of the five), there was a higher risk of D2 among both sexes. As for the substitution of mixed foods, no substitution of them reduced the risk of the disease. Finally, the researchers concluded that, the substitution of pizzas according to the classified categories was associated with a slight decrease in the risk of D2. | Isoenergetic substitutions are more effective in reducing risk, especially when personalized, also non-isoenergetic substitutions present greater uncertainties, highlighting the need for more structured approaches in food reformulation, finally the differences between sexes highlight the importance of considering gender when designing dietary intervention strategies. These findings underline the relevance of promoting healthy dietary substitutions based on optimized nutritional profiles to improve public health outcomes. |
| Burgos et al. [68] | Estimate deaths attributable to excessive sodium consumption in the Paraguayan population in different scenarios and provide subsidies to public health surveillance and decision-makers and stakeholders | For the development of their research, two scenarios were created considering salt reduction, the first of them is the “consumption of less than 5 g of salt per day” and the second is the “30% reduction in the average salt consumption of Paraguayan adults (the national goal for 2024 and the World Health Organization goal for 2030)”. The scenarios were created with the PRIME model, in this way, the impact on health can be evaluated considering the reduction of deaths, incident cases and disability-adjusted life years (DALYs). Also, demographic, mortality, age and sex information available from the National Institute of Statistics of Paraguay was used. Regarding disease information, the Global Burden of Disease study was used, considering coronary heart disease classified according to the ICD-10. In addition, reference data on salt consumption were used from a previous study, considering an average in-take of 3.4 g ± 0.8 g of salt/day in both sexes (considering an intake of less than 5 g/day), and 10.4 g ± 2.5 g (men) and 9.0 g ± 2.7 g in women (considering reductions of 30% in average salt intake, by sex) in adults. | The reducing salt consumption, either through an absolute limit of 5 g/day or a relative reduction of 30%, can generate substantial benefits in the prevention of cardiovascular diseases in Paraguay. These measures would not only reduce mortality and the incidence of cardiovascular events but would also decrease the burden of disability in the population. These findings underscore the need to implement effective public policies that promote the reduction of salt consumption as a key strategy for the prevention of chronic diseases. |
| Briggs et al. [69] | Describe the PRIMEtime cost-effectiveness model (PRIMEtime CE). | For the development of the research, the following information was considered, firstly, the PRIMEtime model, which has a configuration that allows the development of multistate life table that calculates the effect of change for 14 diet-related risk factors, in addition to taking as a basis the 10 PRIME risk factors. Secondly and in the same way, this new model takes the basis of the PRIME model, related to diet and diseases, considering the same analysis of the information that this model has. As for the creation of the PRIMEtime CE model, it allows estimating the cost-effectiveness of interventions affecting diet and PA. This model has information related to diseases such as coronary heart disease, stroke, diabetes II, cirrhosis and cancers. In terms of data and methods, it contains information related to disease incidences, incidence rates, mortality and case fatality rates, and baseline prevalence derived from the WHO DISMOD II, as well as information related to cohort studies, among other types of studies related to diseases and their population. In addition, this model contains risk factors, where we find parameters (and diseases) related to fruit (coronary heart disease, stroke, lung cancer), vegetables (coronary heart disease, lung cancer), fiber in cereals (coronary heart disease), fiber (breast cancer, colorectal cancer, stomach cancer), red meat (colorectal cancer, stomach cancer, diabetes II), processed meat (colorectal cancer, diabetes II), blood cholesterol (coronary heart dis-ease, stroke), systolic blood pressure (coronary heart disease, stroke), body mass index (coronary heart disease, stroke, diabetes, pancreatic cancer, breast cancer, kidney cancer, liver cancer, liver cirrhosis), diabetes (coronary heart disease, stroke) and PA (coronary heart disease, stroke, diabetes, colorectal cancer, breast cancer). | This new model allowed a direct comparison of the public health policies that have an impact on the diseases described in the article. In this way, the model made it possible to estimate the cost and morbidity of the disease, when considering awareness of the risk factors that affect these diseases. |
| Madia et al. [70] | Examine how changes in diet and lifestyle could have potentially reduced the incidence of dis-eases in South Korea in 2022. | The PRIME model was used, where two counterfactual scenarios were created, the first of which was called “optimal world” assuming that all drinkers and smokers quit these habits. The second scenario was called “risk reduction world” assuming a reduction in alcohol consumption (30%) and a reduction in tobacco consumption (-30%). For the development of these scenarios, to obtain demographic information on the population, the Korean Statistical Information Service for 2022 was used; to assess the burden of disease, the information available from the 2019 Global Burden of Disease (GBD) study was used. In addition, information from the Korea National Health and Nutrition Examination Survey (KNHANES) was used to consult on the consumption of risk factors related to dietary habits, tobacco and alcohol consumption. For information related to annual medical expenses, the 2022 National Insurance Statistical Yearbook was used. | The reduction in tobacco and alcohol consumption in the South Korean population by 2022 allowed them to analyze the impact of the reduction of these risk factors in the population under working age (15 to 19 years), working age (20 to 64 years) and older working age (65 years or older). In this way, it was possible to demonstrate possible improvements in people's health and in the reduction of expenses associated with the treatment of NCDs when reducing or eliminating tobacco or alcohol consumption by the population, producing improvements in people's health in the long term. |
| Saito et al. [71] | Examine the relationship between risky consumption behaviors, noncommunicable diseases, and socioeconomic costs in Japan using the Integrated Preventable Risk Model; the potential impact of healthier lifestyle choices on the incidences and costs of NCDs in 2019 was evaluated. | To meet the objectives, the 2019 population information available at the Ministry of Health, Labor and Welfare of Japan was used. Regarding the incidences of NCDs, the information available from the Global Burden of Diseases of 2019 was used. For information related to risk factors, the Japan National Health and Nutrition Survey (JNHNS) 2019 was used, information was obtained from the Nutrient Intake Survey, such as consumption of fat, fiber, salt, fruit, vegetables, tobacco and alcohol consumption. For the assessment of the economic impact in 2022, the expenditures on medical care per inpatient and outpatient service by age group and disease category in Japan were used. Through the use of PRIME, counterfactual scenarios were developed considering the WHO recommendations, but, in addition, regarding the scenario related to tobacco consumption, a previous study was considered where they simulated the consumption of less harmful tobacco alternatives instead of quitting smoking, assuming that 70% of smokers change their consumption to heated tobacco products (HTP). | By 2019, Japan could have saved $35.1 billion dollars, and a total of 564,000 cases of NCDs could have been avoided, provided that recommendations on healthy food consumption, reduction in alcohol consumption and replacing cigarettes with less harmful alternatives are considered. |
| Espinosa Herrera [72] | Evaluate the potential savings of people who adopt reduced-risk behaviors in Mexico. | For the development of the research, the PRIME model was used, as for the information of the population it was obtained from the Projections of the Population of Mexico of the Federative Entities (CONAPO), for health data related to hospital admissions for specific illness it was obtained from the General Directorate of Health Information (DGIS), also from the National Institute of Statistics and Geography (INEGI) 2016. Also, information from the National Health and Nutrition Survey (ENSANUT) was used for data related to the intake of fiber, fat, salt, fruits and vegetables. Regarding the information related to tobacco and alcohol consumption, it was obtained from the National Survey on Drug, Alcohol and Tobacco Consumption (ENCODAT). For the development of counterfactual scenarios using PRIME, the number of cases of diseases that could be prevented through different patterns of unhealthy food consumption was simulated. In addition, the possibility was generated that people could switch from conventional cigarettes to alternatives containing nicotine (NCAs) of reduced risk. | The promoting healthy eating habits, reducing alcohol consumption and transitioning from conventional cigarettes to less harmful options could lead to healthy behaviors and a reduction in health care costs associated with NCD treatment. |
